# Comparison of Gadolinium-enhanced MRI and ^18^FDG PET/PET-CT for the diagnosis of brain metastases in lung cancer patients: A meta-analysis of 5 prospective studies

**DOI:** 10.18632/oncotarget.16182

**Published:** 2017-03-14

**Authors:** Ye Li, Guanqiao Jin, Danke Su

**Affiliations:** ^1^ Department of Radiology, Cancer Hospital of Guangxi Medical University, Nanning, People's Republic of China; ^2^ Department of Radiation Oncology, Cancer Hospital of Guangxi Medical University, Nanning, People's Republic of China

**Keywords:** lung cancer, brain metastases, magnetic resonance imaging, positron emission tomography, positron emission tomography/computed tomography

## Abstract

**Objective:**

We undertook this meta-analysis to compare the significance of Gadolinium-enhanced MRI and ^18^FDG PET/PET-CT for diagnosing brain metastases of lung cancer patients.

**Results:**

Five articles comprising 941 patients were included for analysis. The sensitivities with 95% confidence interval for PET/PET-CT and MRI were 0.21 (0.13 − 0.32) and 0.77 (95% CI = 0.60 − 0.89), specificities were 1.00 (0.99 − 1.00) and 0.99 (0.97 − 1.00), and the areas under curve were 0.98 (0.96 − 0.89) and 0.97 (0.96 − 0.98).

**Materials and Methods:**

A computerized literature search of studies was conducted in the Pubmed and Embase databases. Meta-analysis methods were used to calculate the sensitivities, specificities, likelihood ratios ratios, diagnostic odd ratios, and areas under summary receiver operating characteristic curves for PET/PET-CT and MRI, respectively.

**Conclusions:**

The analysis suggested Gadolinium-enhanced MRI had higher sensitivity than ^18^FDG PET/PET-CT for the diagnosis of brain metastases in lung cancer. MRI may provide additional information to PET-CT for diagnosing brain metastatic lesions.

## INTRODUCTION

Brain metastases are frequent findings in lung cancer patients, accounting for about 14% of newly diagnosed patients [1–2]. Particularly in lung adenocarcinoma, the rate of brain metastases has been reported to be up to 43% [3]. Lung cancers without distant metastases are potentially curable. Hence, accurate localization of brain metastatic lesions may lead to better selection of curative therapy or palliation.

Pretreatment imaging procedures for the evaluation of brain metastatic lesions in lung cancer patients remain controversial issue. In the guidelines of the European Respiratory Society, brain computed tomography (CT) is recommended in all lung cancer patients with neurological symptoms [4]. However, the diagnostic capability of brain CT in patients without neurological abnormalities is still not clear. The use of 18fluorodeoxyglucose positron emission tomography (^18^FDG PET)-CT is more efficient to detect distant metastases than the use of conventional imaging procedures [5]. However, ^18^FDG PET or PET-CT has limited diagnostic performance in the evaluation of brain metastatic lesions, mainly because of its difficulties in differentiating FDG-avid metastases from the normal surrounding hyper-metabolic parenchyma in brain tissue [6]. Magnetic resonance imaging (MRI) has been put forward as another one-stop-shop imaging technique for M staging of lung cancer patients [7, 8]. Compared with PET-CT, brain MRI has the potential to detect more brain metastatic lesions in lung cancer patients [7–9]. However, the application of a meta-analysis to directly compare the diagnostic capability of PET/PET-CT and MRI for the assessment of brain metastases in lung cancer patients has not been explored. In this study, we conducted a meta-analysis of available studies to systematically assess and compare their abilities for diagnosing brain metastatic lesions of lung cancer patients.

## RESULTS

### Study selection and description

**Figure 1:**
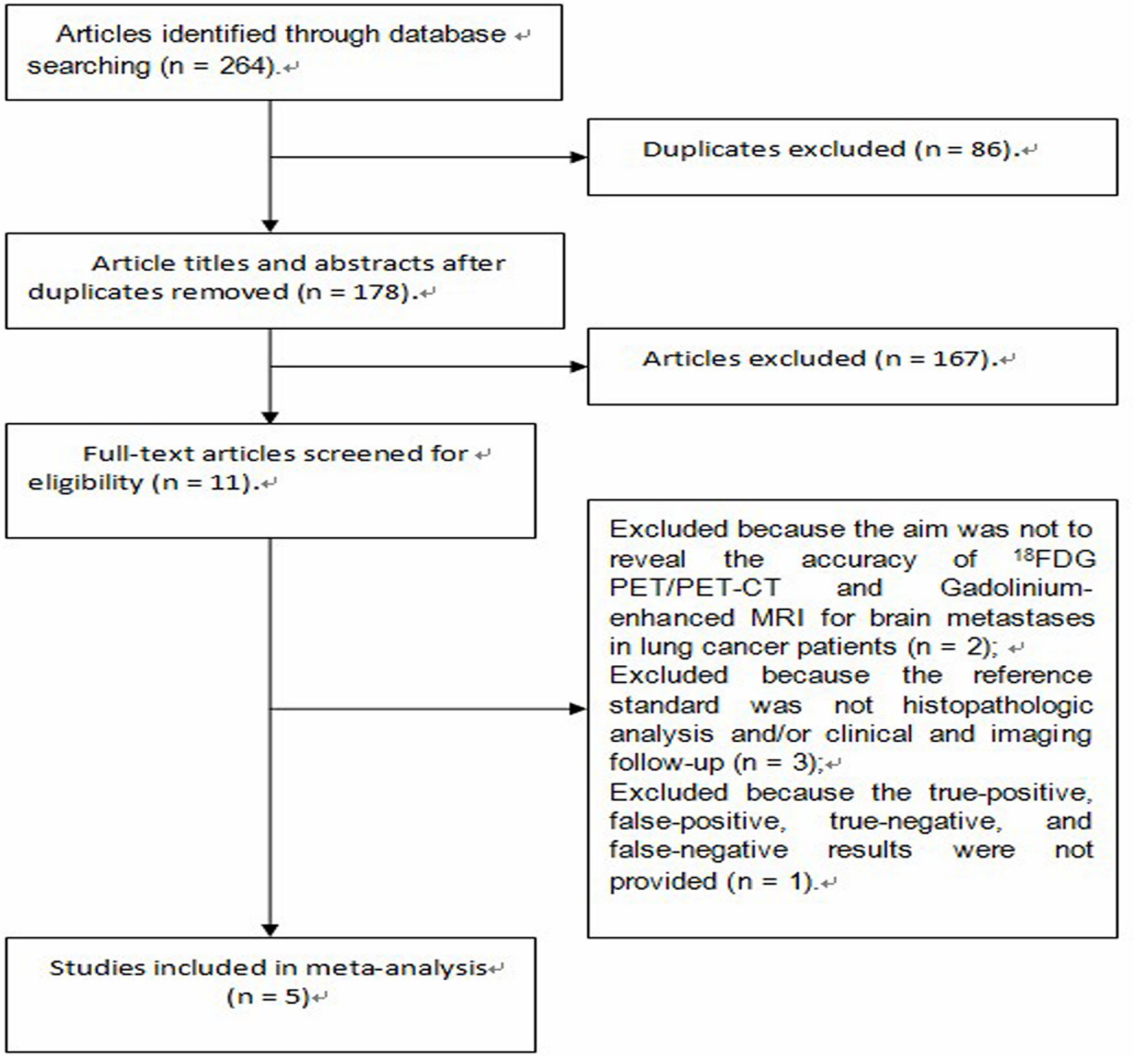
Flow chart of study selection

The electronic search yielded 264 abstracts; Among 264 abstracts, we found that 11 articles were potentially eligible. After we read the full texts of these articles, 6 of the 11 relevant articles were excluded. The search and screening of relevant studies is summarised in Figure 1. Consequently, 5 studies [7, 8, 10–12] involving 941 patients were eligible for this meta-analysis (Figure 1). Characteristics of each eligible study are presented in Table 1. The total number of patients in a single study ranged from 52 to 442 (median, 165 patients). The reported age range was from 23 to 88 years. All studies were of the prospective design. In three studies (307 patients) [7, 8, 10], only non-small cell lung cancer patients were enrolled, in one study (442 patients) [12], only lung adenocarcinoma patients were enrolled, and in the last study (203 patients) [11], all pathological types of lung cancer patients were enrolled.

**Table 1:** The clinical characteristics of ^18^FDG PET/PET-CT and Gadolinium-enhanced MRI

Study	Origin	No. of Patients	Age(y)	Male (%)	Follow-up Time	MRI		PET-CT	
						Strengthen	Sequences	CE-CT	Analysis Methods
Ohno [11], 2007	Japan	90	35–83	53.3	≥ 24 months	1.5T	T1,T2, CE-T1, FLAIR	No	QL
Plathow [10], 2008	Germany	52	49–71	69.2	Unclear	1.5T	T1,T2, CE-T1, STIR	Enhanced by iodinated contrast agent	QL + QN
Yi [7], 2008	Korea	165	34–82	75.8	592 days (mean)	3.0T	T1,T2, CE-T1	No	QL
Ohno [8], 2008	Japan	203	47–85	53.7	≥ 12 months	1.5T	T1,T2, CE-T1, STIR	No	QL
Lee [12], 2009	Korea	442	23–88	53.8	≥ 30 months	3.0T	T1,T2, CE-T1, FLAIR	No	QL + QN

Abbreviations: QL = qualitative; QN = quantitative; CE = contrast enhanced; STIR = short time inversion recovery; FLAIR = fluid-attenuation inversion-recovery.

### Study quality

The results and Criteria of the methodological quality were presented in Table 2. Main disagreements were related to external validity (EV) 5, internal validity (IV) 3, and IV5. All studies had the valid reference tests (IV1). However, the reference tests were based in part on a comparison of initial and follow-up images in all studies [7, 8, 10–12] (IV3). In four studies [7, 8, 10, 12], the interpretation of 18F-FDG PET/CT was conducted without knowing any clinical information (IV5). In three studies [7, 8, 12], eligible patients were enrolled consecutively (EV5).

**Table 2:** Quality assessment of the 5 included articles in this meta-analysis

Study	Internal Validity Criteria						External Validity Criteria						No. of items assessed as “yes” in the criteria
	IV1	IV2	IV3	IV4	IV5	IV6	EV1	EV2	EV3	EV4	EV5	EV6	
Ohno [11], 2007	Yes	Yes	No	Yes	No	Yes	Yes	Yes	Yes	Yes	No	Yes	9
Plathow [10], 2008	Yes	Yes	No	Yes	Yes	Yes	Yes	Yes	Yes	Yes	No	Yes	10
Yi [7], 2008	Yes	Yes	No	Yes	Yes	Yes	Yes	Yes	Yes	Yes	Yes	Yes	11
Ohno [8], 2008	Yes	Yes	No	Yes	Yes	Yes	Yes	Yes	Yes	Yes	Yes	Yes	11
Lee [12], 2009	Yes	Yes	No	Yes	Yes	Yes	Yes	Yes	Yes	Yes	Yes	Yes	11

The Methodological Quality Criteria Recommended by the Cochrane Methods Working Group on Diagnostic Meta-analysis [20].

Internal Validity (IV):

1. Valid reference test (Biopsy, or imaging follow-up);

2. Blind measurement of PET/PET- CT or MRI without knowledge of reference test results;

3. Blind measurement of reference test without knowledge of results of PET/PET-CT or MRI;

4. Assessment by reference test independent of results of PET/PET-CT or MRI;

5. PET/PET-CT or MRI interpreted independently of all clinical information (Mentioned in publication);

6. Prospective study (Mentioned in publication);

External Validity (EV):

1. Spectrum of disease (Primary stage of disease);

2. Demographic information (Age and sex information given);

3. Inclusion criteria (Mentioned in publication);

4. Exclusion criteria (Mentioned in publication);

5. Avoidance of selection bias (Consecutive series of patients);

6. Standard execution of PET/PET-CT or MRI (PET/PET-CT: Type of camera, dose of 18F-fluorodeoxyglucse, time interval, reconstruction; MRI: Strength, dose of contrast medium, sequences, reconstruction);

### Summary estimates of sensitivity, specificity, DOR, PLR, and NLR

#### ^18^FDG PET/PET-CT

The chi-square values of sensitivity, specificity, diagnostic odds ratio (DOR), positive likelihood ratio (PLR), and negative likelihood ratio (NLR) for ^18^FDG PET/PET-CT were 7.18 (*p* = 0.066 > 0.05), 1.39 (*p* = 0.709 > 0.05), 0.81 (*p* = 0.848 > 0.05), 0.23 (*p* = 0.973 > 0.05), and 2.73 (*p* = 0.435 > 0.05), respectively. The pooled sensitivity, specificity, DOR, PLR, and NLR values for ^18^FDG PET/PET-CT were 0.21 (95% confidence interval [CI] = 0.13 to 0.32), 1.00 (95% CI = 0.99 to 1.00), 235 (95% CI = 31 to 1799), 184.7 (95% CI = 24.8 to 1374.0), and 0.79 (95% CI = 0.70 to 0.89), respectively.

#### Gadolinium-enhanced MRI

The chi-square values of sensitivity, specificity, DOR, PLR, and NLR for Gadolinium-enhanced MRI were 7.87 (*p* = 0.097 > 0.05), 11.02 (*p* = 0.026 > 0.05), 2.08 (*p* = 0.026 < 0.05), 2.80 (*p* = 0.591 > 0.05), and 11.08 (*p* = 0.026 < 0.05), respectively. The pooled sensitivity, specificity, DOR, PLR, and NLR values for Gadolinium-enhanced MRI were 0.77 (95% CI = 0.60 to 0.89), 0.99 (95% CI = 0.97 to 1.00), 657 (95% CI = 112 to 3841), 149.6 (95% CI = 24.5 to 913.1), and 0.23 (95% CI = 0.12 to 0.43), respectively.

### Summary receiver operating characteristic curves

**Figure 2:**
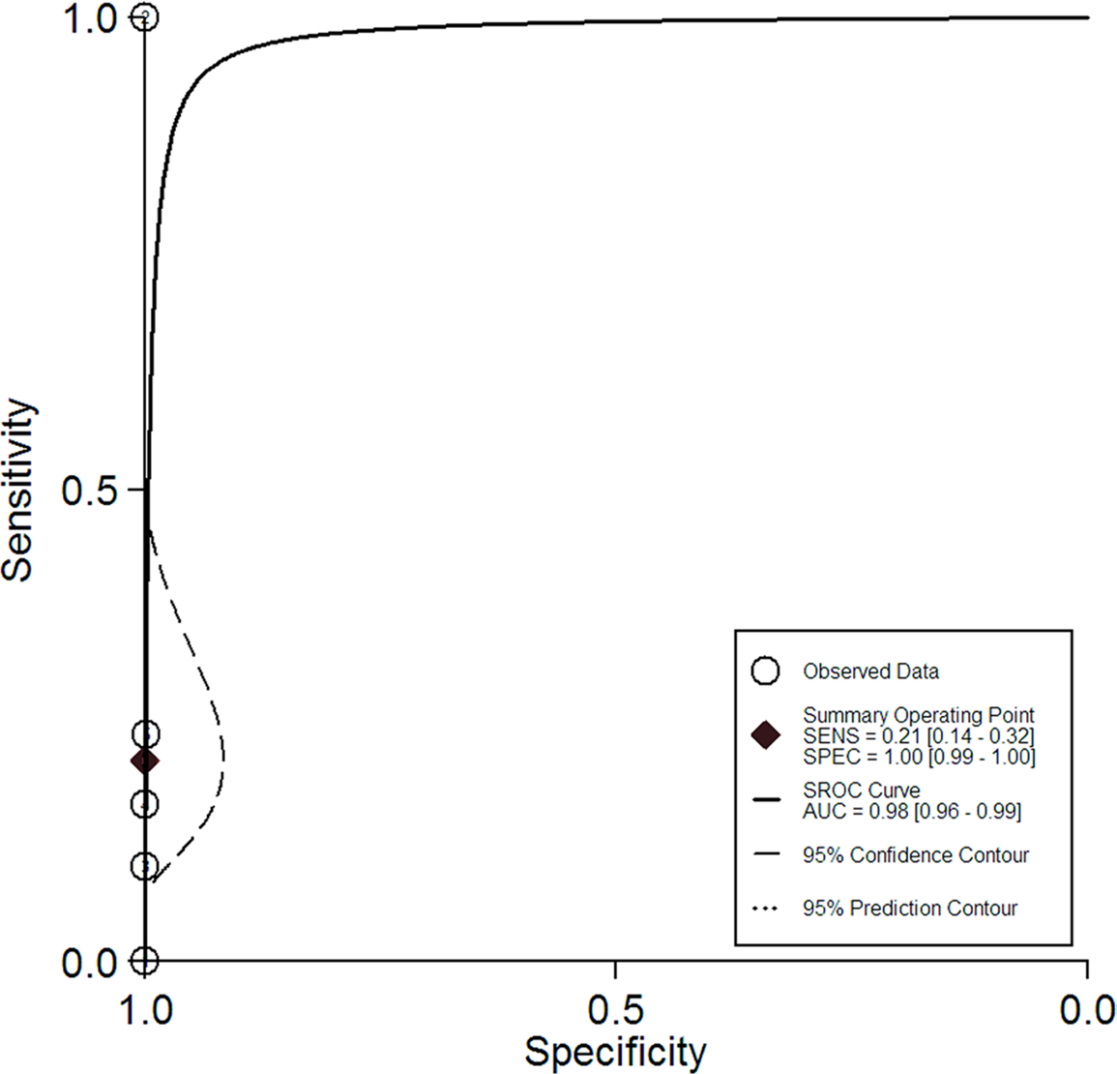
The summary receiver operating characteristic curve for the diagnostic performance of ^18^FDG PET/PET-CT

**Figure 3:**
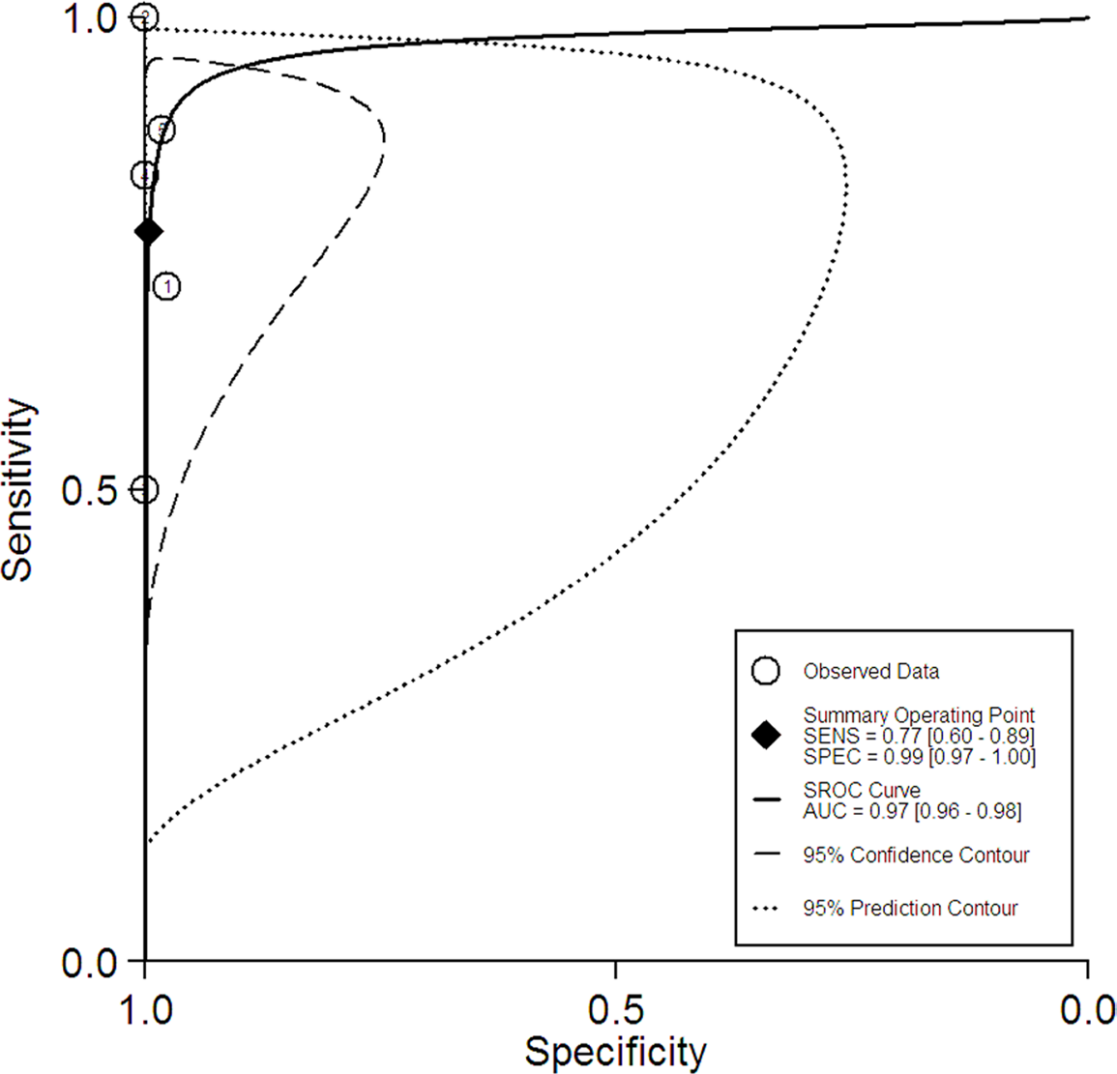
The summary receiver operating characteristic curve for the diagnostic performance of Gadolinium-enhanced MRI

The summary receiver operating characteristic (SROC) curves for ^18^FDG PET/PET-CT and Gadolinium-enhanced MRI were shown in Figures 2 and 3. The areas under the curve were 0.98 (95% CI = 0.96 to 0.89) and 0.97 (95% CI = 0.96 to 0.98), respectively.

## DISCUSSION

The brain is one of the most frequent distant-sites of lung cancer. Jena et al [13] reported that brain metastases accounted for 35.4% of patients with lung cancer (90% patients with IV-stage disease). In another study [14], brain metastases occurred in 11% of 442 lung cancer patients (28% patients with IV-stage disease). The median survival time of lung cancer patients with untreated brain metastatic lesions is less than three months, whereas the median survival time of lung cancer patients with brain metastases receiving palliative radiotherapy is about eight months [14, 15]. The brain is often the only site of distant metastatic disease [16]. Precise assessment of brain metastatic lesions can offer more opportunities to act early and elicit a better therapeutic effect.

Now the differences of the efficacy between PET/PET-CT and MRI for the assessment of brain metastatic lesions were still controversial. In this study, we obtained summary estimates and SROC curves for the clinical value of PET/PET-CT and MRI. ^18^FDG PET/PET-CT has limited diagnostic performance for the assessment of brain metastatic lesions. Gadolinium-enhanced MRI has higher sensitivity (77% *vs* 21%) than ^18^FDG PET/PET-CT. Gadolinium-enhanced MRI should be performed additionally to PET-CT for additional information to PET-CT in lung cancer patients with a curative option. It may, however, be noted that the high specificity of a positive PET/PET-CT finding may provide important clinical information in a setting where the brain is routinely included in the PET/PET-CT scan and contraindications for the apply of MRI.

The DOR is a single metric of test accuracy that combines sensitivity and specificity into a single number [17]. The higher value of DOR indicates better discriminatory test performance. The pooled DOR values for ^18^FDG PET/PET-CT and Gadolinium-enhanced MRI in this study were 235 and 657, indicating a higher level of accuracy for these two modalities. Likelihood ratios are also the indicators that take into account the interaction between the sensitivity and the specificity in their calculation. The values of PLR > 10 and NLR < 0.1 are considered convincing evidence to rule in or rule out disease [18, 19]. The pooled PLR values of for ^18^FDG PET/PET-CT and Gadolinium-enhanced MRI were 184.7 and 149.6, which were therefore high enough to diagnose brain metastatic lesions of lung cancer. The pooled NLR values for ^18^FDG PET/PET-CT and Gadolinium-enhanced MRI were 0.79 and 0.23, indicating that the negative results of these two modalities couldn't be used alone to exclude brain metastatic lesions of lung cancer.

In this study, we searched with a systematic search strategy, selected available studies according to the strict criteria of inclusion, and assessed the methodological quality using uniform criteria. All these steps can increase the reliability of the results. However, several inevitable limitations must also be addressed when interpreting the results of this meta-analysis. First, imaging follow-up was used as one part of the reference standard in all studies. It might not correctly classify brain metastatic lesions in some patients with a refusal of biopsy. Besides, some parameters (such as pathological type, staging, diagnosis standards, glucose, radiotracer dose and uptake period of PET) were not considered in our study because of incomplete data. This may affect the accuracy of these two modalities. Third, publication bias was not tested because the few number of included studies may induce potential bias. Fourth, the MRI data in 4 of the 5 available studies is from the whole-body MRI procedures. The MRI technique of brain used in such whole-body protocols is probably not the most efficient protocol for the assessment of brain metastatic lesions. This may decrease the real sensitivity of gadolinium-enhanced MRI, which is compared to brain MRI examinations fully dedicated to brain metastatic lesions. Nevertheless, this will not change the final conclusion of this meta-analysis.

In conclusion, Gadolinium-enhanced MRI has higher sensitivity than ^18^FDG PET/PET-CT for the assessment of brain metastatic lesions in lung cancer patients. Gadolinium-enhanced brain MRI examinations may provide some additional information to ^18^FDG PET-CT for definitive exclusion of brain metastatic lesions.

## MATERIALS AND METHODS

### Literature search

A comprehensive literature search was performed to identify articles about the diagnostic capacity of ^18^FDG PET/PET-CT and Gadolinium-enhanced MRI for the assessment of brain metastatic lesions in lung cancer patients. The MEDLINE and EMBASE databases (last update December 2016) were used for searching relevant articles with the following combination of search terms: PET, “positron emission tomography”, MRI, “magnetic resonance imaging”, “distant metastases”, staging, “brain metastases”, NSLC, SLC, AND “lung cancer”. The sample search strategy is presented in Table 3. We had no language restrictions for searching and identifications relevant studies. To expand our search, references of relevant articles were screened for potentially suitable studies.

**Table 3:** The search strategy used for the MEDLINE and EMBASE databases

#	Search string
1	PET OR “positron emission tomography”
2	MRI OR “magnetic resonance imaging”
3	“distant metastases” OR staging OR “brain metastases”
4	NSLC OR SLC OR “lung cancer”
5	#1 AND #2 AND #3 AND #4

### Study selection

Studies comparing the accuracy of ^18^FDG PET/PET-CT and Gadolinium-enhanced MRI for the assessment of brain metastatic lesions in lung cancer patients were eligible for inclusion. Studies with the data on a per-patient analysis were included. Studies with only one imaging modality (PET/PET-CT or MRI) were excluded. Review articles, editorials, abstracts, case reports, and guidelines for management and studies with less than ten participants were excluded. Studies were excluded if brain metastases were not confirmed by histopathologic analysis and/or imaging follow-up. Studies that didn't provide sufficient data to construct a 2 × 2 contingency table for the calculation of sensitivity and specificity were also excluded. When data were presented in more than one article, the article with the largest number of patients or the article with the most details was chosen. Studies in which PET/PET-CT and MRI were not performed within one month of one another were also excluded.

Two reviewers (L.L, GQ.J) independently reviewed the titles and abstracts of the retrieved articles, applying the inclusion and exclusion criteria mentioned above. Articles were rejected if they were clearly ineligible. The same two reviewers (L.L, GQ.J) then independently reviewed the full-text version of the remaining articles to determine their eligibility for inclusion. Disagreements were resolved in consensus meetings.

### Data extraction

Two reviewers (L.L, GQ.J) independently extracted the relevant data from each article and recorded these data on a standardized form. And any disagreement was resolved in consensus meetings. Data was extracted from the studies, including study authors, publication time, study design, number of participants, and imaging technical characteristics of ^18^FDG PET/PET-CT or Gadolinium-enhanced MRI, the reference standard, and totals of true positives, false positives, true negatives, and false negatives.

### Quality assessment

We assessed the methodological quality of the included studies using the criteria list recommended by the Cochrane Methods Working Group on Diagnostic Meta-analysis [20]. Some items on the list for internal validity (IV) and external validity (EV) were modified for this meta-analysis (Table 2). Every criteria was assessed as “Yes” or “No”.

### Statistical analysis

Data on the diagnostic performance of PET/PET-CT and MRI were combined quantitatively across eligible studies. The degree of heterogeneity among eligible studies was reported using the chi-square statistic. The threshold for significance in this chi-square statistic was defined as *p* < 0.05. We calculated the sensitivities, specificities, positive likelihood ratios (PLR), negative likelihood ratios (NLR), and diagnostic odds ratios (DOR) with 95% confidence intervals (CI) for PET/PET-CT and MRI using the bivariate model [21]. This bivariate model allows for more between- and within-study variability than do the fixed-effect models. We also constructed the SROC curves to show the summary trade-off between sensitivity and specificity across the eligible studies and calculated the areas under curve for PET/PET-CT and MRI, respectively [22]. All analyses were performed using Stata version 12.0 (Stata Corporation, College Station, TX).
